# Acetate Causes Alcohol Hangover Headache in Rats

**DOI:** 10.1371/journal.pone.0015963

**Published:** 2010-12-31

**Authors:** Christina R. Maxwell, Rebecca Jay Spangenberg, Jan B. Hoek, Stephen D. Silberstein, Michael L. Oshinsky

**Affiliations:** 1 Department of Neurology, Thomas Jefferson University, Philadelphia, Pennsylvania, United States of America; 2 Department of Pathology, Anatomy and Cell Biology, Thomas Jefferson University, Philadelphia, Pennsylvania, United States of America; Institute of Cellular and Developmental Biology, Greece

## Abstract

**Background:**

The mechanism of veisalgia cephalgia or hangover headache is unknown. Despite a lack of mechanistic studies, there are a number of theories positing congeners, dehydration, or the ethanol metabolite acetaldehyde as causes of hangover headache.

**Methods:**

We used a chronic headache model to examine how pure ethanol produces increased sensitivity for nociceptive behaviors in normally hydrated rats.

**Results:**

Ethanol initially decreased sensitivity to mechanical stimuli on the face (analgesia), followed 4 to 6 hours later by inflammatory pain. Inhibiting alcohol dehydrogenase extended the analgesia whereas inhibiting aldehyde dehydrogenase decreased analgesia. Neither treatment had nociceptive effects. Direct administration of acetate increased nociceptive behaviors suggesting that acetate, not acetaldehyde, accumulation results in hangover-like hypersensitivity in our model. Since adenosine accumulation is a result of acetate formation, we administered an adenosine antagonist that blocked hypersensitivity.

**Discussion:**

Our study shows that acetate contributes to hangover headache. These findings provide insight into the mechanism of hangover headache and the mechanism of headache induction.

## Introduction

Alcohol hangover headache is one of the most common types of headache, yet the mechanism of how ethanol causes headache pain is unknown [1], [2]. Both migraine sufferers and non migraineurs experience hangover headache, but migraine sufferers experience more severe hangover headaches with less alcohol consumption [3]. Hangover headache (veisalgia cephalgia) or delayed alcohol induced headache occurs from four to twenty four hours after the end of drinking and can cause migraine-like symptoms including unilateral throbbing pain with photophobia in migraineurs [3]. Animal models of headache recreate the tactile sensitivity associated with headache but the mechanism of headache induction remains elusive [4], [5]. The current study employs a rat model of recurrent headache to examine the mechanism of alcohol induced pain.

Most clinical studies of hangover have been retrospective, questionnaire-based studies and do not address the pathophysiological mechanism by which ethanol causes headache [6], [7]. Due to the lack of controlled mechanistic studies, there is a wide variety of theories concerning the origin of veisalgia cephalgia. Ethanol is metabolized into acetaldehyde by alcohol dehydrogenase, mainly in the liver, and then converted into acetate by mitochondrial aldehyde dehydrogenase in many tissues, including brain tissue [8], [9], [10]. Most reviews suggest that acetaldehyde is responsible for the hangover [11], [12], [13]. Markedly increased circulating acetaldehyde levels after alcohol consumption may cause vasodilation, flushing of the face, nausea, and headache in approximately one third of people originating from East Asian countries who are homozygous or heterozygous for a defective form of aldehyde dehydrogenase-2 (ALDH-2), [8], [14], [15], [16]. In addition, disulfiram, an aldehyde dehydrogenase inhibitor, given with ethanol, causes symptoms similar to those seen in individuals expressing functionally inactive ALDH-2. This indirect evidence suggests that increased acetaldehyde concentrations may induce headaches. The caveat is that the serum concentration of acetaldehyde when disulfiram is combined with ethanol is much higher than that achieved after ethanol ingestion alone [14]. Serum acetaldehyde concentrations with ethanol alone are very low in normal individuals because acetaldehyde is rapidly metabolized into acetate [17], [18], [19]. This suggests that the headache experienced after the combination of ethanol and disulfiram and in individuals who lack functional ALDH-2 is not due to the same mechanism as the hangover headache experienced in the absence of disulfiram.

Although serum and urine acetaldehyde levels are low and change minimally following ethanol intake, acetate levels are significantly elevated for at least six hours [18], [20]. Studies suggesting that acetaldehyde is responsible for hangover have not considered acetate, which increases to much higher levels (millimolar) in the circulation, even after moderate drinking, compared to acetaldehyde levels (micromolar) [12], [13], [18]. Acetate alone may induce headaches at these elevated concentrations [21]. This is supported by the clinical observation that acetate given during kidney dialysis causes headache [22].

Studies report that dehydration, impurities, and congeners (compounds that can increase the taste or smell of the beverage) may also play a role in hangover headache induction [23], [24]. Dehydration is a migraine trigger in some people, and increased water intake has been proposed as a preventive treatment [25]. Reports suggest that more severe hangover symptoms result following intoxication with brandy than with vodka, suggesting that congeners exacerbate the hangover but not necessarily the headache [12], [24]. Red and white wine contain sulfites, tannins, phenols, and other compounds that are thought to cause hangover headache by increasing plasma levels of serotonin and histamine [26], [27], [28]. The concomitant consumption of foods high in serotonin (bananas and pineapples) may exacerbate headaches [29]. Although congeners may worsen the hangover headache, there are no studies demonstrating that they trigger pain.

This study uses a rat model of recurrent headache in which repeated infusions of inflammatory soup (IS) onto the dura increase cutaneous sensitivity to mechanical stimuli on the periorbital region of the face [5]. This mimics the human condition of repeated activation of the trigeminal nociceptive pathway resulting in periorbital tactile sensitivity. Similar to human migraineurs, these *sensitized* rats are hypersensitive to chemical headache triggers such as nitroglycerin [5].

We examined the effects of ethanol, a dietary headache trigger, and its metabolites in our animal model of recurrent headache. Ethanol metabolism was manipulated by pharmacological inhibition of ethanol metabolizing enzymes. The results of our study point to acetate as the critical metabolite of ethanol in the induction of delayed alcohol induced headache. The mechanism for the induction of headache following increased serum levels of acetate may be through a downstream effect of acetate such as the accumulation of adenosine. These findings challenge the conventional theories of the mechanism of hangover headache induction.

## Methods

Male Sprague Dawley rats (250–300 g, Charles River n = 84) were individually housed in a temperature-controlled environment, under a 12-hour light/dark cycle, and allowed access to food and water ad libitum.

### Ethics Statement

All procedures performed on the animals were approved by the Thomas Jefferson University Institutional Animal Care and Use Committee, animal welfare assurance number A3085-01, protocol numbers 685S and 685T. All surgeries were performed under isoflurane anesthesia. All efforts were made to minimize animal number and suffering.

### Implantation of the cannula

Surgical procedures were previously described [5]. Briefly, after allowing two weeks for habituation and training, animals were fitted with plastic caps and stainless steel cannulae (26 gauge, Plastics One Inc., Roanoke, VA, USA). Rats were put under isoflurane anesthesia (3% induction, 1.5% maintenance) mixed with compressed air. A ∼3 mm wide craniotomy was performed just above the junction of the superior sagittal and transverse sinuses to expose the dura over the transverse sinus along the midline. The cannula was placed over the opening and secured to the skull with a combination of small screws and dental cement. The cannula was then sealed with an obdurator that was custom cut to extend just beyond the internal end of the cannula above the dura. This prevented scar tissue from forming that would eventually obstruct the flow of compounds onto the dura. Rats were allowed one week to recover, during which trigeminal pressure thresholds were monitored to ensure that thresholds were at their presurgery baseline.

### Infusion of Inflammatory Soup or Saline

To infuse the IS, rats were placed in a square plexiglass chamber (17.5×18×12 inches) that allowed for free movement during the infusion of IS or saline onto the dura. The IS contained 1-mM histamine, serotonin, bradykinin, and 0.1 mM prostaglandin E2 in 0.9% sterile saline [5]. Polyethylene tubing (PE50) was connected to a microinfusion pump (WPI Inc, Sarasota, FL, USA) as well as to the exposed cannula. This pump allowed for a steady infusion of 25 µl of IS or saline over 5 minutes at a rate of 5 µl per minute while the rats moved freely. Tactile sensory testing, described below, was performed before and at several time points after infusion. The obdurators were replaced after the infusion. Our laboratory has previously shown that IS infusions 3 times/week over the course of three weeks (8 to 9 infusions) causes the rats to transition into a sensitive state where they are susceptible to compounds including nitroglycerin, which can induce headaches in migraineurs [30].

Post mortem procedures verified cannula placement on top of the dura. The cannula was flushed to ensure it was not clogged and a visual inspection was done on the dura to ensure correct placement over the junction of the superior sagittal and transverse sinuses.

### Tactile Sensory Testing

Rats, tested during the day, were trained and acclimated to a plastic tube restraint (inner diameter 8 cm, length 25 cm) before and after cannula implantation, and entered uncoaxed. This restrainer prevented the rats from walking away from the sensory testing.

Periorbital pressure thresholds were determined by applying von Frey monofilaments (Stoelting Co., Wood Dale, IL, USA) to both the left and right sides of the face over the rostral portion of the eye. These von Frey hairs are calibrated nylon monofilaments that generate a reproducible buckling stress. Each monofilament is identified by manufacturer assigned force values (10, 8, 6, 4, 2, 1.4, 1, 0.6, 0.4, 0.16, 0.07 grams). The higher the force value on the monofilament, the stiffer and more difficult it is to bend. For each time point, the left and right threshold data are recorded separately. The von Frey stimuli were presented in sequential order, either ascending or descending, as necessary, to determine the threshold of response (modified from [31]). After a positive response, a weaker stimulus was presented, and after a negative response, a stronger stimulus was presented. The threshold is defined as a positive response to 2 of 3 trials of the von Frey monofilament. Results are presented either as the threshold in grams +/− SEM, or as a percent change from baseline on the side that had the lowest value. Several behaviors presented by the rat were considered a positive response. The rat would vigorously stroke its face with the ipsilateral forepaw, quickly recoil its head away from the stimulus, or vocalize. Rats that did not respond to the 10 g stimulus were assigned 10 g as their threshold for analysis.

### Experimental Groups

The main objective of this study is to demonstrate that ethanol induces periorbital hypersensitivity in an animal model of repeated trigeminal stimulation. Pharmacological manipulations have been used to dissect which step in the ethanol metabolism cascade is responsible for this effect. The experimental groups are listed below:

1. Ethanol Treatment alone or combined with the following:
2. 4-Methyl Pyrazole- inhibits alcohol dehydrogenase, preventing the formation of acetaldehyde.
3. Disulfiram- inhibits aldehyde dehydrogenase, preventing the formation of acetate.
4. Acetate- major metabolite of ethanol (administered alone).
5. Caffeine- adenosine antagonist, used because increased serum acetate levels lead to an accumulation of adenosine in the brain.
6. Ketorolac- anti-inflammatory drug used to treat headache.

#### Ethanol treatment

Forty eight to seventy two hours after the 8^th^ infusion of either IS or saline, rats were administered ethanol (300 mg/kg) or control (2.5 ml) liquid diet (Bio-Serv, Frenchtown, NJ, USA) via oral gavage, creating four animal groups in this experiment. The liquid diet, administered to ensure absence of congeners and to promote normal hydration throughout the experiment, consisted of 18% of the total calories from protein, 35% from fat, 11% from carbohydrates and 36% from ethanol [32]. Rats received a single administration of control or ethanol liquid diet and had free access to water and standard rodent chow throughout the duration of all experiments. There were six rats in each animal group except the group that received saline infusions and control diet (n = 5). Tactile sensory thresholds were recorded prior to and following control or ethanol treatment hourly for six hours. Hydration levels were monitored using the skin pinch dehydration test. All rats had normal skin elasticity throughout the study, indicating proper hydration.

#### Locomotor activity

To address the possibility that our dose of ethanol can reduce motor activity, we recorded motor activity for one hour following ethanol and control gavage. The ethanol dose was 300 mg/kg and the control volume was 2.5 ml. Each rat (n = 8) received both ethanol and control conditions, administered 48 to 72 hours apart with order of administration randomized. Locomotor activity was recorded in a square plexiglass chamber (17.5×18×12 inches) with infrared beams across the bottom and two sides (Med Associates, St. Albans, VT, USA). Infrared beam breaks were recorded on a computer and total distance traveled in 60 minutes was analyzed in 10 minute epochs.

#### Ketorolac Treatment

Since ethanol causes decreased thresholds in IS treated rats, we examined the ability of ketorolac (Bedford Laboratories, Bedford, OH, USA), a non-steroidal anti-inflammatory drug, to prevent this effect. Because there were no significant effects of ethanol on infusion naive rats, ketorolac was only given to IS infused rats with ethanol (300 mg/kg). Rats (n = 4) received either an intraperitoneal (i.p.) injection of saline (0.5 ml) or ketorolac (0.4 mg/kg) one hour after ethanol administration [33]. These rats received ethanol twice, once before a saline injection and then at least 72 hours later, before a ketorolac injection.

#### 4-Methyl Pyrazole

4-methyl pyrazole (Sigma-Aldrich, St Louis, MO, USA) prevents the conversion of ethanol to acetaldehyde by inhibiting alcohol dehydrogenase or CYP2E1 [34], [35]. As ethanol is ingested, it accumulates without conversion to acetaldehyde or acetate through these pathways. The combination of ethanol (300 mg/kg) and 4-methyl pyrazole (50 mg/kg) was administered via oral gavage to 5 rats that received IS infusions [34]. Thresholds were monitored hourly for at least six hours. There is no persistent sensitivity in saline infused or infusion naive rats and no identifiable analgesic response, therefore this drug was only administered to rats that received IS infusions.

#### Disulfiram

Disulfiram (Sigma-Aldrich, St Louis, MO, USA) irreversibly inactivates aldehyde dehydrogenase, preventing the conversion of acetaldehyde to acetate and causing an accumulation of acetaldehyde [14], [17], [19]. The combination of ethanol (300 mg/kg) and disulfiram (100 mg/kg) was administered via oral gavage to 5 rats that received IS infusions [36]. Our laboratory has previously shown that there are no significant differences in threshold between saline infused rats and infusion naive rats. The control rats for this set of experiments are infusion naive [5]. An additional 4 infusion naive rats received the same combination of ethanol and disulfiram. Thresholds were monitored hourly for six hours.

#### Acetate Treatment

We tested the effects of acetate (Sigma-Aldrich, St Louis, MO, USA) on thresholds in both IS infused and infusion naive rats in the absence of ethanol treatment. Sodium acetate (20 and 60 mg/kg i.p) was administered to IS infused and infusion naive rats (n = 5 per group). Thresholds were monitored hourly for six hours. Sodium acetate salt was administered at a pH of 7 buffered in physiological saline to avoid gut inflammation and visceral pain.

#### Serum Acetate Measurements

Serum acetate was measured in untreated rats and rats that received either 60 mg/kg acetate or 300 mg/kg ethanol (n = 2 per group). Trunk blood was collected one hour after administration of drugs or no treatment and centrifuged for serum. Serum was then centrifuged using an Amicon ultra-4 centrifugation filter (Millipore, Billerica, MA) at 4000xg for 30 minutes (modified from [37]). Filtrate was collected and acetate was measured by manufacturer's specifications using acetic acid test combination UV- at 340 nm (Boehringer/R-Biopharm Marshall, MI).

#### Caffeine

The adenosine antagonist, caffeine (50 mg/kg, Sigma-Aldrich, St Louis, MO, USA), was administered i.p. three hours after ethanol gavage (300 mg/kg) to rats with a history of repeated IS infusions (n = 5) [38]. A second set of IS infused rats (n = 4) were given caffeine (50 mg/kg i.p.) to control for the effects of caffeine on IS infused rats in the absence of ethanol. Thresholds were monitored hourly.

### Statistical Analyses

#### Ethanol

**Figure 1:**
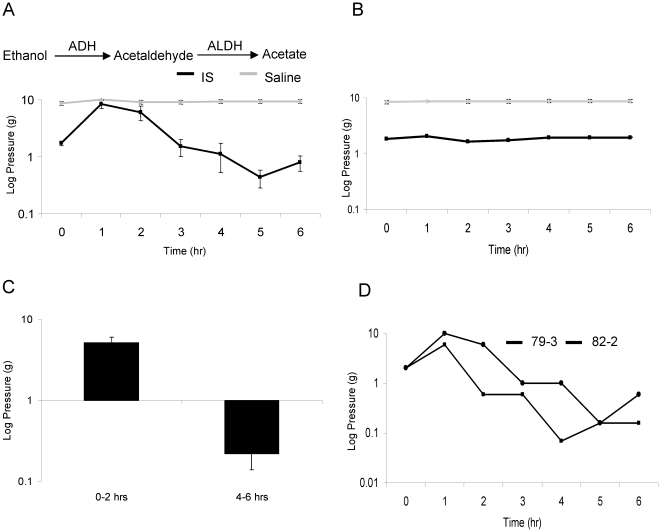
Ethanol induces a biphasic change in periorbital tactile sensitivity. Figure 1a displays an ethanol-induced biphasic change in tactile sensitivity that is specific to the rats infused with IS, also referred to as sensitized rats. Figure 1b shows no change in threshold with administration of control gavage to IS (black) and saline (grey) infused rats. Figure 1c illustrates the change in threshold relative to baseline at 0–2 hours following ethanol and 4–6 hours following ethanol. Figure 1d demonstrates the temporal variability of the biphasic response to ethanol in two individual rats that had previously been infused with IS. Note logarithmic scale.

The variability in the time points at which thresholds changed in response to ethanol was accounted for in the statistical analyses (Fig. 1). A repeated measures ANOVA was used to compare baseline thresholds to the maximum value from 0 to 2 hours following treatment and the minimum value from 4 to 6 hours, creating 3 repeated factors per animal. Significant interactions of time, infusion condition (IS or saline) and ethanol condition (ethanol or control diet) are decomposed in the results section using SPSS v.12 (IBM Co, Chicago, IL, USA). Difference scores or calculating the difference between two time points to give one statistical value per rat, were also used to decompose interactions for ethanol and other drug conditions throughout the study. LSD post hoc was used for significant ANOVA findings throughout the study.

#### Ketorolac

Minimum threshold values from 4 to 6 hours in the saline condition were compared to the corresponding time point during ketorolac treatment. Difference scores between baseline and aforementioned values were compared within animals using repeated measures ANOVA.

#### Locomotor activity

Total distance traveled across 60 minutes was first normalized to the average distance traveled per session per rat to reduce variability, and then binned into ten minute epochs. T-tests were performed to compare the ethanol session and the control session at each epoch for a total of 6 comparisons.

#### 4-Methyl Pyrazole

Baseline values were compared to the maximum value from 0 to 2 hours and the minimum value from 4 to 6 hours creating 3 repeated factors per animal. Animals that received 4-methyl pyrazole + ethanol were compared with those that received ethanol alone (from ethanol treatment study) to minimize the number of animals used. A univariate analysis of variance was used to compare 4-methyl pyrazole + ethanol with ethanol alone at baseline, 0 to 2 hours post gavage and 4 to 6 hours post gavage.

#### Disulfiram

As in the 4-methyl-pyrazole analysis, IS infused rats that received disulfiram + ethanol were compared to ethanol alone (from ethanol treatment study) at baseline, 0 to 2 hours, and 4 to 6 hours following gavage.

Infusion naive rats administered disulfiram + ethanol were compared with saline infused rats that received ethanol alone to minimize the number of animals used. A univariate analysis of variance was used to compare disulfiram + ethanol to ethanol alone at baseline, 0 to 2 hours post gavage and 4 to 6 hours post gavage.

#### Acetate

Statistical analysis was performed using a repeated measures ANOVA for the individual groups of rats, comparing baseline sensory thresholds to the minimum threshold from 1 to 4 hours following each dose of acetate within each rat. The minimum threshold was analyzed because of the temporal variability seen in these rats following ethanol administration.

#### Caffeine

A repeated measures ANOVA was used to compare IS infused rats that received ethanol alone with IS infused rats that received an injection of caffeine at 3 hours after ethanol administration. Time was the repeated measure and this was a between-animals design. Since the ethanol time course following caffeine seemed to be shifted compared with ethanol alone, a repeated measures ANOVA was performed comparing baseline and time points following caffeine administration within animals that had received both ethanol and caffeine. An additional repeated measures ANOVA was performed comparing baseline and the four 1 hour time points following caffeine in IS infused rats that received caffeine in the absence of ethanol.

## Results

### Ethanol induces a biphasic change in sensory thresholds only in sensitized rats

Sensitized rats are defined as those that received repeated infusions of IS and demonstrated baseline thresholds of 2 grams or less; normal rats have thresholds of 8–10 grams. Ethanol treatment via gavage caused analgesia in the form of decreased sensitivity for nociceptive behaviors for the first two hours (p<0.001), followed by increased pain sensitivity from 4 to 6 hours only in sensitized rats (p = 0.001) (Fig. 1a–d).

In non sensitized rats, control or ethanol gavage caused no significant differences in threshold across time (p = 0.465 and p = 0.25, respectively). In the sensitized rats that received control gavage, there were also no significant changes in threshold (p = 0.444). However, in the sensitized rats that received ethanol gavage, significant differences in threshold were found across the three time points (p<0.001).

### 4-Methyl pyrazole, an alcohol dehydrogenase inhibitor, extends the duration of the analgesic response

**Figure 2:**
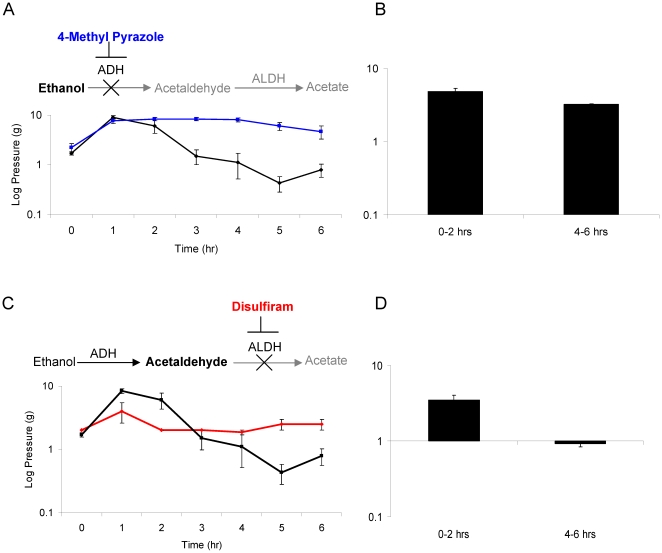
Inhibition of alcohol metabolism by alcohol dehydrogenase or acetaldehyde metabolism by aldehyde dehydrogenase cause analgesia and prevents nociceptive behaviors. Figure 2a illustrates changes in threshold over 6 hours in IS infused rats that received ethanol combined with 4-Methyl Pyrazole (4MP blue line). Thresholds are also shown for ethanol alone (black line) from Figure 1a as a comparison. Figure 2b displays change in threshold relative to baseline at 0–2 hours following ethanol +4MP and 4–6 hours following ethanol +4MP. Figure 2c illustrates changes in threshold over time in IS infused rats that received ethanol combined with disulfiram (red line). Thresholds for ethanol alone (black line) are included from Figure 1a as a comparison. Figure 2d displays change in threshold relative to baseline at 0–2 hours following ethanol + disulfiram and 4–6 hours following ethanol + disulfiram.

4-Methyl pyrazole increased the duration of ethanol induced analgesia for more than 4 hours after gavage (p<0.001) (Fig. 2a–b) and prevented the ethanol induced hypersensitivity during the 4 to 6 hour time period. No differences were found between ethanol alone and ethanol combined with 4-methyl pyrazole in sensitized rats at baseline (p = 0.312) or within two hours following ethanol exposure (p = 0.166).

### Disulfiram, an aldehyde dehydrogenase inhibitor, shortens the analgesic response

Disulfiram shortened the duration and magnitude of the analgesia observed in sensitized rats during the first two hours after ethanol administration, but did not significantly change thresholds from 4 to 6 hours after treatment compared with baseline (Fig. 2c, d). From 0–2 hours, sensitized rats administered the combination had lower thresholds than the ethanol alone group (p = 0.003). From 4 to 6 hours, disulfiram and ethanol produced higher thresholds than ethanol alone (p<0.001). No differences were found at baseline (p = 0.111).

Four infusion naive rats were administered the combination of ethanol and disulfiram. No significant differences in threshold were found between these animals and saline infused rats administered ethanol alone (baseline: p = 0.393; 0–2 hrs p = 0.999; 4 to 6 hrs p = 0.861).

### Acetate contributes to ethanol induced hypersensitivity

**Figure 3:**
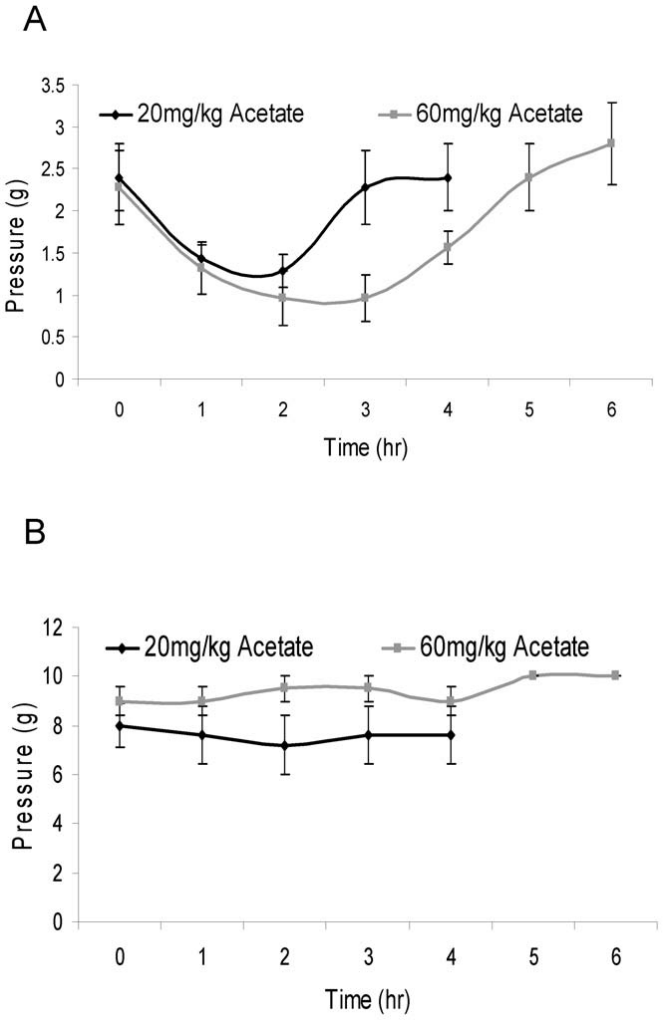
Acetate causes ethanol induced hypersensitivity. Figure 3a displays changes in threshold for sensitized rats that received 20 (black) and 60 (grey) mg/kg acetate i.p. Figure 3b shows changes in threshold for infusion naive rats that received 20 (black) and 60 (grey) mg/kg acetate.

Within three hours following acetate administration (20 and 60 mg/kg), sensitized rats had a significant decrease in sensory threshold (p = 0.024 and 0.023), while infusion naive rats showed no decrease at either dose (p = 0.178 and p = 0.99) (Fig. 3a–b). Acetate increased the magnitude and duration of nociceptive behaviors in a dose-dependent manner.

Circulating serum acetate levels were measured in rats treated with 60 mg/kg acetate, 300 mg/kg ethanol or no treatment. Untreated rats had serum acetate concentrations of 0.3 mM reflecting endogenous serum levels of acetate. Both ethanol and acetate treated rats had serum acetate concentrations of approximately 1 mM (0.96 and 1.09 mM respectively).

### Caffeine blocks ethanol induced hypersensitivity

**Figure 4:**
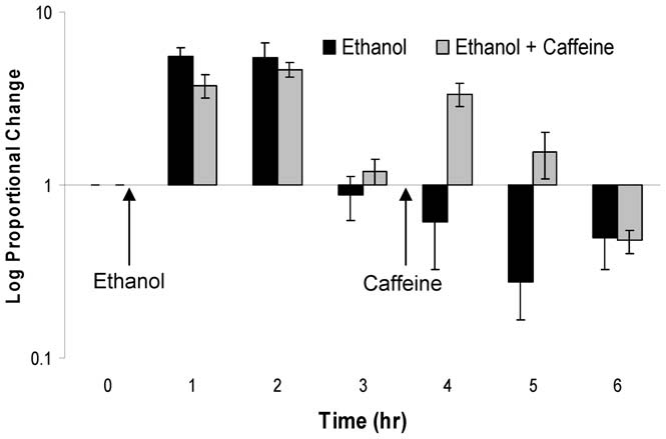
Caffeine blocks ethanol induced hypersensitivity. Figure 4 shows the effect of caffeine (i.p.) on periorbital pressure thresholds 3 hours after ethanol (gavage) administration. The black bar depicts ethanol alone. The grey bar shows time course following the combination of ethanol and caffeine.

Sensitized rats were treated with ethanol, and subsequently given an i.p. injection of caffeine 3 hours after ethanol administration. Caffeine was given three hours after ethanol administration to test if it will prevent the increased sensitivity seen 4 to 6 hours following ethanol. Significant differences were found between caffeine and ethanol alone at four (p<0.001) and five (p = 0.011) but not six (p = 0.467) hours after ethanol administration (Fig. 4). Since the combined treatment of ethanol and caffeine produced thresholds that decreased below baseline 5 to 7 hours after ethanol administration (which differs from the 4 to 6 hours seen with ethanol alone), a second ANOVA revealed significant differences between baseline at this later time point (p = 0.001), indicating that caffeine blocks ethanol induced hypersensitivity. However, once caffeine is metabolized (t_1/2_ = 1.2 hrs in rats), the hypersensitivity returns [39]. Caffeine administered to sensitized rats in the absence of ethanol had no effect on thresholds across 4 hours (p = 0.775).

### Ketorolac, an NSAID, prevents ethanol induced hypersensitivity

**Figure 5:**
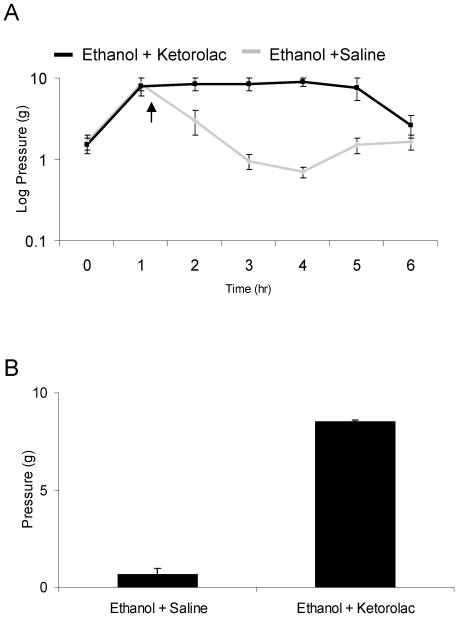
Ketorolac blocks ethanol induced hypersensitivity. Figure 5a demonstrates the effect of ethanol on periorbital pressure thresholds when given an injection of saline or ketorolac. Figure 5b shows the minimum thresholds between 4 and 6 hours post ethanol in rats that were given ethanol + saline or ethanol + ketorolac.

Sensitized rats were treated with ethanol via oral gavage and subsequently given an i.p. injection of either saline or ketorolac one hour after ethanol administration (Fig. 5a–b). Ketorolac reversed the decrease in threshold seen with ethanol treatment (p = 0.011), but had no effect on ethanol induced analgesia.

### Low dose ethanol does not influence locomotor activity

Locomotor activity was measured for one hour following ethanol or control gavage. A t-test revealed no significant differences in activity across 60 minutes between groups given control and 300 mg/kg of ethanol (Table 1).

**Table 1:** Motor activity during ethanol and control treatment.

Time (min)	Difference in activity	Standard error	t	Significance (2-tailed)
0–10	311	381	0.82	0.441
11–20	333	229	1.45	0.189
21–30	−411	237	−1.73	0.127
31–40	−150	238	−0.63	0.548
41–50	332	165	2.00	0.085
51–60	−415	255	−1.63	0.147

Table 1 shows the difference in locomotor activity between control and ethanol gavage in ten minute epochs. Positive numbers represent higher activity rates in the control condition. Negative numbers represent higher activity rates in the ethanol condition. T-test results are included.

## Discussion

While animal models of headache have been able to recreate migraine symptoms, including tactile sensitivity, modeling headache induction is still elusive. This novel demonstration of inducible headache like pain using a dietary headache trigger represents an important step for characterizing mechanisms and developing therapeutics. We show the effects of ethanol and its two major metabolites, acetaldehyde and acetate, in an animal model of trigeminal pain. Ethanol initially decreased nociceptive behaviors and sensitivity. This was followed by increased sensitivity to trigeminal mechanical stimuli. The initial decrease in nociceptive behaviors is due to ethanol induced analgesia. In the 19^th^ century, ethanol was used during human surgery as an anesthetic (summarized in [40]). Ethanol induces analgesia in many rat nociceptive behaviors, including tail flick and cold water test at doses ranging from 0.5 to 1.5 g/kg [41]. In addition, Friedman and colleagues found analgesia in other behaviors such as shock induced startle, vocalizations and overt movements in rats [42]. Similar behavioral phenotypes were found in mice given ethanol [43]. Ethanol induced analgesia is mediated through the GABA neurotransmitter system [44], [45]. Molecular studies have suggested a role for the G-protein coupled inwardly rectifying potassium channels in analgesia [44]. While the ethanol induced analgesia is well documented in the literature, little is known regarding its pronociceptive properties.

Ethanol induced delayed trigeminal hypersensitivity 4 to 6 hours after administration, when blood ethanol levels are expected to be close to zero [46], [47], [48]. The decrease in periorbital pressure threshold is analogous to the mechanical allodynia seen in patients during a headache [4]. Ketorolac reversed this decrease in threshold, suggesting that the rats have inflammatory pain. Interestingly, the decrease in threshold was only found in sensitized rats with a prior history of repetitive trigeminal nociceptor stimulation. This is in consensus with clinical studies that show an increase in hangover vulnerability in patients with a history of recurrent headaches [1], [2], [3]. Low dose ethanol, equivalent to approximately one standard drink in humans, was chosen to differentiate between sensitized rats and those naive to trigeminal pain. In humans, a delayed alcohol induced headache (DAIH) is characterized by pain occurring from 4 to 24 hours following ingestion and is correlated with blood ethanol levels at or returning to zero [3]. In addition, migraineurs can experience DAIH with a modest intake of alcohol, whereas non migraineurs require larger amounts to produce the phenomenon [3]. Therefore, our findings model the sensitivities seen in migraineurs.

Most authors claim that acetaldehyde is responsible for hangover symptoms, but they neglected to consider the rapid formation of acetate from acetaldehyde [12], [13]. Our results provide direct evidence that acetate, not acetaldehyde, contributes to the delayed increase in trigeminal sensitivity. Ethanol plus disulfiram, which is known to increase acetaldehyde levels, did not produce delayed hypersensitivity for nociceptive behaviors. This suggests that acetaldehyde may not directly contribute to the pain seen in our model. Alternatively, the low dose of ethanol may not have been sufficient to produce a toxic build up of acetaldehyde when given with disulfiram. Disulfiram at 100 mg/kg provides approximately 80% inhibition of aldehyde dehydrogenase 1 and 2 and 61% inhibition of aldehyde dehydrogenase 3 in the rat [36]. Since a majority of aldehyde dehydrogenase was inhibited by disulfiram and headache pain is reported with the combination of disulfiram and ethanol in alcoholics, acetaldehyde accumulation did not cause the pain seen in our model.

Acetate administration induced an immediate decrease in trigeminal pressure thresholds in sensitized rats. The dose of acetate administered (60 mg/kg) produces serum acetate levels of 1.09 mM in rats, which is equivalent to acetate levels attained with our dose of ethanol (0.95 mM) and consistent with previously published data in rats [49] and humans [18]. Thus, we used physiologically relevant doses of acetate to induce increased sensitivity for nociceptive behaviors, indicating that acetate may contribute to DAIH. The similar effects of ethanol and acetate have been shown in behaviors including motor coordination and sensitivity to anesthesia [49]. Patients experience headaches when acetate is used as a buffer for kidney dialysis at serum concentrations ranging from 2 to 4 mM, but headache history and their susceptibility to headache was not measured [22].

These data do not completely exclude the role of acetaldehyde in hangover. Although treatment with ethanol in the presence of disulfiram did not induce a headache in our sensitized rats, these data provide indirect evidence that acetaldehyde may not be involved in ethanol induced trigeminal pain. Acetaldehyde may still cause hangover symptoms in humans including headache. The data in this study demonstrate that acetate also contributes to the headache component of the hangover.

Signaling cascades downstream of acetate can promote pain. Acetate increases adenosine in many tissues, including the brain [49], [50]. The adenosine receptor antagonist, caffeine, administered after ethanol, blocked the nociceptive behaviors associated with ethanol. This suggests that adenosine contributes to ethanol induced hypersensitivity. Caffeine and ketorolac are used to treat hangover symptoms in humans. Non-steroidal anti-inflammatory drugs (ketorolac) alleviate the headache pain associated with alcohol hangovers and caffeine is indicated to ease other hangover symptoms, including fatigue and malaise [7], [12], [13].

Our findings challenge the concept that dehydration and congeners alone are the cause of hangover headache. Our rats were normally hydrated and administered pure ethanol. While dehydration and congeners may contribute to the hangover in humans, this is the first demonstration that acetate accumulation may play a role in hangover headache induction.

We have shown that ethanol, a migraine trigger in humans, lowers nociceptive threshold in sensitized rats with a history of trigeminal nociceptor stimulation. This is similar to the effect of nitroglycerin, a nitric oxide donor, both clinically and in our model [5]. Nitroglycerin induces a short dull headache in non migraineurs but in migraine patients can trigger a long lasting migraine attack [51]. In naive rats, nitroglycerin causes a decrease in threshold for nociceptive behaviors for about 30 minutes. However, in rats with repeated dura stimulation, nitroglycerin administration produces significantly decreased thresholds for more than two hours [5]. Our model is thus sensitive to two common triggers of headache, nitric oxide donors and ethanol. Interestingly, the timing of delayed nociception in our model allows for future studies of the molecular mechanism of headache induction with ethanol or other triggers.

Many studies have used inflammatory dura stimulation to model different types of headaches. Migraine, a primary headache disorder, is characterized by sensitivity to light, sound and touch on the head and face. Facial allodynia is sensitivity to innocuous stimuli that is mediated by the trigeminal neurovascular system [4], [52]. Reports from various laboratories indicate that inflammatory stimulation of the dura causes allodynia on the face in animals, through convergence of dura and periorbital afferents at the level of the trigeminal system [5], [53], [54], [55]. This phenomenon is consistent with human data [4], [56]. Our laboratory has also shown increased sensitivity to sound or phonophobia following repeated dura stimulation, which has been demonstrated in humans both during and between migraine attacks [57], [58], [59]. As described in the current study, our model is sensitive to common headache triggers including ethanol and nitroglycerin.

Ethanol in sensitized rats induces analgesia, followed by increased sensitivity for nociceptive behaviors, modeling delayed alcohol induced headache. Acetate contributes to the pain portion of the ethanol hangover, in part through adenosine receptors. This is the first demonstration of a potential mechanism for ethanol hangover headache.
